# Porcine Granulosa-Cell-Derived Exosomes Enhance Oocyte Development: An In Vitro Study

**DOI:** 10.3390/antiox13030348

**Published:** 2024-03-14

**Authors:** Jiajie Ren, Yue Ding, Junsong Shi, Shengchen Gu, Lvhua Luo, Zhihao Feng, Ting Gu, Zheng Xu, Sixiu Huang, Zicong Li, Zhenfang Wu, Gengyuan Cai, Linjun Hong

**Affiliations:** 1 State Key Laboratory of Swine and Poultry Breeding Industry, National Engineering Research Center for Breeding Swine Industry, College of Animal Science, South China Agricultural University, Guangzhou 510000, China; 976991864@stu.scau.edu.cn (J.R.); dingyue@stu.scau.edu.cn (Y.D.); shengchen_gu@stu.scau.edu.cn (S.G.); tinggu@scau.edu.cn (T.G.); stonezen@scau.edu.cn (Z.X.); sxhuang815@scau.edu.cn (S.H.); lizicong@scau.edu.cn (Z.L.); wzf@scau.edu.cn (Z.W.); 2 Guangdong Provincial Key Laboratory of Agro-Animal Genomics and Molecular Breeding, South China Agricultural University, Guangzhou 510000, China; zhihaofeng@stu.scau.edu.cn; 3 Yunfu Subcenter of Guangdong Laboratory for Lingnan Modern Agriculture, Yunfu 527300, China; junsongstone@stu.scau.edu.cn (J.S.); luolvhua@wens.com.cn (L.L.); 4 Key Laboratory of South China Modern Biological Seed Industry, Ministry of Agriculture and Rural Affairs, Guangzhou 510000, China

**Keywords:** pigs, oocyte, granulosa cells, exosomes, miR-148a-3p, antioxidants

## Abstract

Recent studies have established that exosomes (EXs) derived from follicular fluid (FF) can promote oocyte development. However, the specific sources of these EXs and their regulatory mechanisms remain elusive. It is universally acknowledged that oocyte development requires signal communication between granulosa cells (GCs) and oocytes. However, the role of GC-secreted EXs and their functions are poorly understood. This study aimed to investigate the role of porcine granulosa-cell-derived exosomes (GC-EXs) in oocyte development. In this study, we constructed an in vitro model of porcine GCs and collected and identified GC-EXs. We confirmed that porcine GCs can secrete EXs and investigated the role of GC-EXs in regulating oocyte development by supplementing them to cumulus–oocyte complexes (COCs) cultured in vitro. Specifically, GC-EXs increase the cumulus expansion index (CEI), promote the expansion of the cumulus, alleviate reactive oxygen species (ROS), and increase mitochondrial membrane potential (MMP), resulting in improved oocyte development. Additionally, we conducted small RNA sequencing of GC-EXs and hypothesized that miR-148a-3p, the highest-expressed microRNA (miRNA), may be the key miRNA. Our study determined that transfection of miR-148a-3p mimics exerts effects comparable to the addition of EXs. Meanwhile, bioinformatics prediction, dual luciferase reporter gene assay, and RT-qPCR identified DOCK6 as the target gene of miR-148a-3p. In summary, our results demonstrated that GC-EXs may improve oocyte antioxidant capacity and promote oocyte development through miR-148a-3p by targeting DOCK6.

## 1. Introduction

The ovary is the central organ in the reproductive system of female mammals, with the follicle being its fundamental functional unit [1]. The fertility of sows is directly affected by follicular development, which, in turn, impacts the economic efficiency of pig farming. Due to the persistent atresia and apoptosis of primordial follicles [2], female fertility decreases with age. This is primarily due to oocyte ageing and mitochondrial dysfunction in the surrounding cells, as well as a decrease in antioxidant capacity [3]. Therefore, it is critical to improve oocyte development. The mature follicle comprises oocytes, granulosa cells (GCs), follicular membrane cells, and follicular fluid (FF) [4]. FF contains various factors that influence oocyte development, including nutrients, trace elements, hormones, cytokines, and exosomes (EXs) [5], all of which exert distinct effects on physiological processes, including the proliferation and apoptosis of GCs, oocytes, and follicular membrane cells [6]. GCs surround oocytes and are primarily responsible for transforming estrogen and synthesizing progesterone [7]. GCs are essential for oocyte maturation and development. Healthy follicle growth and survival cannot be achieved without communication and signaling between GCs and oocytes [8]. Follicular formation and development are intricate processes that are subject to a combination of autocrine, paracrine, and endocrine regulatory effects. In recent years, exosomes derived from follicular fluid (FF-EXs) have been discovered to have a profound impact on the reproductive function of animals [9,10,11]. Gabryś [12] demonstrated that FF-EXs promote equine cumulus expansion and increase the maturation rate of oocytes. However, the sources of FF are very complex and predominantly include plasma osmolates, GC secretions, and theca cell secretions [13]. Likewise, the sources of FF-EXs and their regulatory roles in the reproductive system microenvironment remain unclear. Furthermore, it is worthwhile emphasizing that the majority of the existing studies have concentrated on FF-EXs, with a dearth of research on porcine granulosa-cell-derived exosomes (GC-EXs).

EXs are vesicles with a diameter of 30–150 nm and a bilayer membrane structure. They are secreted by cells and are extensively found in animal body fluids. EXs carry various substances, such as mRNA, microRNA (miRNA), and proteins [14,15]. Under a transmission electron microscope (TEM), EXs exhibit a cup-like structure. Moreover, they are stored in multivesicular bodies (MVBs). When MVBs fuse with the plasma membrane of the cell, EXs are released extracellularly [16]. The released EXs can deliver and transfer substances to recipient cells upon contact [17]. Among these molecules, miRNA has garnered considerable attention owing to its role in regulating gene expression [18].

MiRNA is a type of endogenous non-coding RNA composed of 20–23 nucleotides and can bind to the 3′ UTR fragment of mRNA to inhibit mRNA expression [19]. MiRNA plays a pivotal role in cells and can also be released into the external environment by cells through various packaging forms [20]. It is abundant in the animal reproductive system and participates in regulating various reproductive processes, including spermatogenesis, oocyte maturation and expulsion, fertilization, embryo development, and embryo implantation [21]. Some miRNAs carried by EXs can participate in gene regulation and signaling pathways related to follicular development [22,23], thereby regulating the proliferation and apoptosis of GCs, as well as oocyte meiosis and maturation. Furthermore, miRNAs present in bovine FF-EXs are implicated in regulating the signaling pathway for follicle development and oocyte growth.

In order to investigate the specific source of FF-EXs, we shifted our research perspective to investigate whether GCs can produce EXs, if they do, and the effect of EXs on oocyte development. In this study, porcine GC-EXs were isolated and collected for the first time, and our experimental results confirmed that GC-EXs and miR-148a-3p exert an important effect on porcine reproductive capacity, thereby promoting oocyte development and maturation and concomitantly alleviating oxidative stress. Our results provide valuable insights for improving oocyte development and enhancing porcine oocyte culture in vitro. Meanwhile, they also provide clues for improving porcine fertility and potential clinical applications for the treatment of female ovarian diseases.

## 2. Materials and Methods

### 2.1. Acquisition of Porcine FF

Healthy porcine ovaries were obtained at a local slaughterhouse and transported to the laboratory at 37 °C. As previously described [24], the ovaries were cleaned using normal saline containing 1% penicillin–streptomycin(Gibco, Grand Island, NY, USA) at 37 °C. Follicles with a bright appearance, normal development, and multiple full follicles with a cavity and diameter ranging between 3–8 mm were selected. The FF was aspirated with an 18-gauge needle from 20 selected pairs of ovaries.

### 2.2. Isolation and Culture of Porcine Ovarian GCs

As previously described [25], the FF should be centrifuged at 500× *g* for 5 min at room temperature. The supernatant was discarded, and the precipitate was centrifuged three times. Then, the precipitate was resuspended with DMEM/F12 (Gibco, Grand Island, NY, USA) supplemented with 1% penicillin–streptomycin–gentamicin solution (Beyotime, Shanghai, China) and 10% fetal bovine serum (Gibco, Grand Island, NY, USA) and subsequently inoculated into a 100 mm cell-culture dish that was inserted in a 5% CO_2_ cell incubator at 37 °C. Once the cells adhered to the wall, the medium was changed. The previous studies have reported that fetal bovine serum contains EXs, which may affect the collection of EXs in cellular supernatants [26]. Therefore, EX-free serum was used in this study. Given that GCs are terminally differentiated cells, their morphology and function may change after three generations. Therefore, only the culture medium of the second- and third-generation GCs was collected for EX isolation.

### 2.3. Isolation and In Vitro Culture of Porcine Oocytes

The FF was allowed to stand for 20 min. The supernatant was then discarded, and DPBS (Gibco, Grand Island, NY, USA) containing 1% penicillin–streptomycin (Gibco, Grand Island, NY, USA) was added at 37 °C. After resuspension, the supernatant was discarded, and this process was repeated three times. Uniform-sized cumulus–oocyte complexes (COCs) with clear and uniform cytoplasm and dense, complete cumulus cells were selected. As previously described [27], the COCs were transferred to 30 mm cell-culture dishes containing oocyte maturation medium comprising Medium 199 (Sigma-Aldrich, Burlington, MA, USA); the medium was supplemented with 10% EXs-free fetal bovine serum (*v*/*v*), 10% follicular fluid (*v*/*v*), 10 IU/mL human chorionic gonadotropin (hCG), 10 ng/mL epidermal growth factor (EGF), 10 IU/mL pregnant mare serum gonadotropin (PMSG), and 0.1 mg/mL L-cysteine and washed three times. Next, they were randomly grouped and cultured in an incubator at 38.5 °C, 5% CO_2_, and saturated humidity for 42–44 h.

### 2.4. Preparation of EX-Free Fetal Bovine Serum

As previously described [26], upon thawing the fetal bovine serum at 4 °C, it was centrifuged at 160,000× *g* in an ultracentrifuge (Beckman Coulter Instruments, Fullerton, CA, USA) for 12–16 h. Then, the supernatant was transferred to a new centrifuge tube, and the precipitate was discarded. The EX-free fetal bovine serum was obtained by filtration through a 0.22 μm filter to remove impurities.

### 2.5. Isolation of GC-EXs

The EXs were isolated from the supernatant of GCs culture solution using a combination of differential centrifugation and ultracentrifugation. As previously mentioned [28], the culture supernatant was successively centrifuged at 300× *g* for 10 min, 2000× *g* for 10 min, and 10,000× *g* for 60 min. After each centrifugation, the supernatant was removed, and the precipitate was discarded. The remaining supernatant was then filtered using a 0.22 μm filter. The supernatant was discarded after ultracentrifugation at 120,000× *g* for 2 h. The centrifugation process was repeated twice to collect the precipitate. All centrifugation steps were performed at 4 °C. The GC-EXs suspension was obtained using 100 μL of pre-cooled DPBS and stored at −80 °C.

### 2.6. Identification by Transmission Electron Microscopy (TEM)

The EXs were identified using transmission electron microscopy [29] (Talos F200S, FEI, Waltham, MA, USA). The GC-EXs suspension was thawed on ice. Next, 10 μL of suspension droplets were absorbed onto a carbon-coated copper mesh and left to react at room temperature for 2 min. Next, the excess liquid was absorbed using filter paper and left to dry. A 3% uranium acetate solution was dropped onto the copper mesh, and the sample was stained for 2 min.

The collected GCs were fixed overnight at 4 °C. They were then treated with a 1% osmic acid solution for 1–2 h, followed by dyeing with a 1% uranyl acetate solution overnight. After rinsing four times with ddH_2_O, the samples were dehydrated using ethanol solutions (30%, 50%, 70%, 85%, and 95%) and then treated with anhydrous ethanol twice for 15 min each time. Finally, pure acetone was used twice for 20 min each time. The sample was treated with a mixture of acetone and embedding agent (*V*/*V* = 3/1; 1/1; 1/3) for 2–8 h. After 24 h of treatment with the pure embedding agent, the sample was embedded and heated at 70 °C overnight. Afterward, the embedded sample was sliced using a Leica UC7 ultra-thin microtome to obtain 70–90 nm slices. Finally, the slices were stained with uranium dioxyacetate solution and lead citrate solution for 15–30 min before being observed under a TEM.

### 2.7. Nanoparticle Tracking Analysis (NTA)

The GC-EXs were thawed on ice. As previously described [30], the sample pool was cleaned three times with ultra-pure water and the instrument was calibrated. Next, 1–2 μL of the sample was absorbed into 2 mL DPBS and mixed well. The diluted sample was then added to the sample pool and analyzed using ZetaView (Particle Metrix, Meerbusch, Germany). The results were plotted using GraphPad Prism 9.0.

### 2.8. Protein Extraction and Protein Concentration Determination

The total protein was extracted from the EXs using RIPA Lysis Buffer (CWBIO, Beijing, China) and incubating on ice for 15 min [31]. The lysate was then collected and centrifuged at 12,000 rpm for 5 min at 4 °C. The resulting supernatant was mixed with protein loading buffer and heated at 100 °C for 10 min to denature the protein. The protein concentration was measured following the instructions provided with the Micro BCA Protein Assay Kit (CWBIO, Beijing, China).

### 2.9. EXs Labeling and Delivery Analysis

Using the previously described method [32], the EXs were stained by diluting them with 1 mL of diluent and adding 4 μL of PKH67 (Sigma-Aldrich, St. Louis, MO, USA). After a 4 min incubation period, 2 mL of 0.5% BSA was added to the mixture. The EXs with green fluorescence were obtained by centrifuging at 120,000× *g* for 2 h. The 100 μg/mL EXs stained with PKH67 were then incubated with COCs. After incubation, the culture medium was discarded, and the samples were washed with DPBS three times. The samples were then fixed in 4% paraformaldehyde (Shyuanye, Shanghai, China) for 10 min. After fixation, the cells were permeabilized with Triton X-100 (Beyotime, Shanghai, China) for 10 min. Finally, TRITC Phalloidin (YEASEN, Shanghai, China) was added, and the resulting mixture was incubated in the dark for 30 min. Finally, the DAPI dye was added and incubated for 10 min. The fluorescence imaging was performed using laser confocal microscopy (Zeiss, Oberkochen, Germany).

### 2.10. Porcine Oocyte Maturation and Cumulus Expansion

After culturing COCs for 42–44 h, the cumulus expansion index (CEI) was calculated based on the COCs morphology, as previously described [33,34]. The cumulus expansion of COCs was divided into five degrees: degree 0 indicates no cumulus expansion; degree 1 reflects an expansion confined within the outermost layer of 1–2 GCs; degree 2 indicates a radial expansion of the outer GCs, resulting in relatively fluffy COCs; degree 3 indicates that only the rest of the oocyte expanded, while the corona radiata did not; degree 4 indicates that all cumulus cells have expanded. The CEI value was calculated using the following formula: CEI = [(number of degree 0 oocytes * 0) + (number of degree 1 oocytes * 1) + (number of degree 2 oocytes * 2) + (number of degree 3 oocytes * 3) + (number of degree 4 oocytes * 4)]/total number of oocytes.

COCs that had been incubated for 42–44 h were placed into a 1.5 mL centrifuge tube containing 0.3 mg/mL of hyaluronidase (HAE). The oocytes were classified as immature (without the first polar body) or at metaphase II (with the first polar body), and their number was recorded. The oocyte maturation rate(the proportion of oocytes reaching metaphase II stage) = the number of oocytes containing the first polar body/the total number of oocytes after degranulation.

### 2.11. Detection of Mitochondrial Membrane Potential (MMP) Levels in Porcine Oocytes

According to the manufacturer’s instructions, the Mitochondrial Membrane Potential Assay Kit with TMRE (Beyotime, Shanghai, China) was used to detect the levels of MMP [35]. Briefly, the mature oocytes were washed with DPBS and incubated in TMRE dye solution in a dark environment for 40 min. After incubation, the oocytes were washed with DPBS three times and visualized under a fluorescence microscope (ZEISS, Oberkochen, Germany). There were eight replicates in each group, with eight oocytes per replicate. The intensity of fluorescence was measured by Image J (1.54 i) software.

### 2.12. Detection of Reactive Oxygen Species (ROS) Levels in Porcine Oocytes

According to the manufacturer’s instructions, the Reactive Oxygen Species Assay Kit (Beyotime, Shanghai, China) was used to detect the levels of ROS [36,37]. In brief, the mature oocytes were selected and washed with DPBS. They were then placed in a 10 μM DCFHDA dye solution and incubated at 38 °C in the dark for 20 min. After incubation, the oocytes were washed with DPBS three times and visualized under a fluorescence microscope. There were eight replicates in each group, with eight oocytes per replicate. The intensity of fluorescence was measured by Image J (1.54 i) software.

### 2.13. RNA Extraction and Analysis of Gene Expression by Real-Time Quantitative PCR (RT-qPCR)

The total RNA was extracted from porcine GC-EXs using the miRNeasy Serum/Plasma Kit (Qiagen, Hilden, Germany) following the manufacturer’s instructions. After 42–44 h of culture, the COCs were transferred into a 1.5 mL centrifuge tube containing HAE, and the GCs and oocytes were collected separately. The RNA was extracted from the GCs and oocytes using the HiPure Total RNA Nano Kit (Magen, Guangzhou, China) following the manufacturer’s instructions.

The HiScript III All-in-one RT Super Mix Perfect for qPCR (Vazyme, Nanjing, China), Mir-X miRNA First-Strand Synthesis and TB Green qRT-PCR User Manual (TaKaRa, Tokyo, Japan), and miRNA 1st Strand cDNA Synthesis Kit (by stem-loop) (Vazyme, Nanjing, China) were employed according to the manufacturer’s instructions. Three biological replicates were performed in two technical replicates [38]. Using GAPDH as the internal control, cel-miR-39 as the external standard, and U6 as the internal standard, respectively, fluorescence quantitative PCR was performed to determine the relative expression levels of genes, and the results were calculated according to the 2^−ΔΔCt^ method [39]. The primer sequence information is presented in Table S1.

### 2.14. Small RNA (sRNA) Sequencing and Data Analysis

The sRNA libraries were constructed following the manufacturer’s instructions using the NEBNext Multiplex Small RNA Library Prep Set for Illumina (Illumina, San Diego, CA, USA). The library quality was assessed using the Agilent Bioanalyzer 2100 system. Subsequently, the library preparation was sequenced on the Novaseq 6000 platform, generating 50 bp single-ended reads.

After discarding the adapter and contaminated sequences, clean reads were mapped to the pig reference genome using the BWA package [40]. To identify the conserved miRNAs, the predicted miRNA hairpins were compared against miRNA precursor sequences from miRBase22.0 using mirDeep2 (v.2.0.0.5) [41], and srna-tools-cli (http://srna-tools.cm p.uea.ac.uk, accessed on 15 December 2022) [42] was utilized to identify potential miRNAs and generate secondary structures. The expression level of the known miRNAs was determined by mapping reads to the miRBase database and then estimated by transcript per million (TPM) according to the following formula: Normalized expression = mapped read count/Total reads × 1,000,000. The target genes of miRNAs were predicted using two computational target prediction algorithms (miRanda (v.3.3) [43] and RNAhybrid (v.2.1.2) [44]). Gene ontology (GO) enrichment and KEGG pathway analysis of target genes were performed using the clusterProfiler R package.

### 2.15. miRNA Transfection

Referring to the transfection methodology described in an earlier study [45,46], miR-148a-3p mimics/miRNA mimics NC were diluted in Opti-MEM (Gibco, Grand Island, NY, USA) according to the instructions for Lipofectamine 3000 Transfection Reagent (Invitrogen, Waltham, MA, USA). The final concentration of miRNA was 40 nM. The complex was incubated with Lipofectamine 3000 for 15 min to form, and then added to the culture solution. The synthesis of miR-148a-3p mimics and miRNA mimics NC was performed by GenePharma (Suzhou, China).

### 2.16. Double Luciferase Report Verification

The target genes of miR-148a-3p were predicted using two tools, namely, miRanda and RNAhybrid. To construct wild-type (WT) plasmids, the 3′-UTR region containing DOCK6 and the fragment of the miR-148a-3p binding site were cloned into the Pmir-GLO vector, and the mutant plasmid (MUT) was designed based on the sequence of the wild-type vector. PK-15 cells were co-transfected with miR-148a-3p mimics and vectors using the transfection method described in Section 2.15. After transfection for 24 h, as previously described [47], the Dual Luciferase Reporter Gene Assay Kit (YEASON, Shanghai, China) was used to detect luciferase activity following the manufacturer’s instructions.

### 2.17. Statistical Methods

Three or more biological replicates were used for statistical analysis in all experiments. The data were analyzed and plotted using GraphPad Prism 9.0 software. T-test was used for comparison between groups, and the results were expressed as mean ± SD. In this experiment, *p* > 0.05 meant no significant difference, *p* < 0.05 meant significant difference, and *p* < 0.01 meant extremely significant difference (ns *p* > 0.05, * *p* < 0.05, ** *p* < 0.01, *** *p* < 0.001, and **** *p* < 0.0001).

## 3. Results

### 3.1. Isolation and Identification of GC-EXs

**Figure 1:**
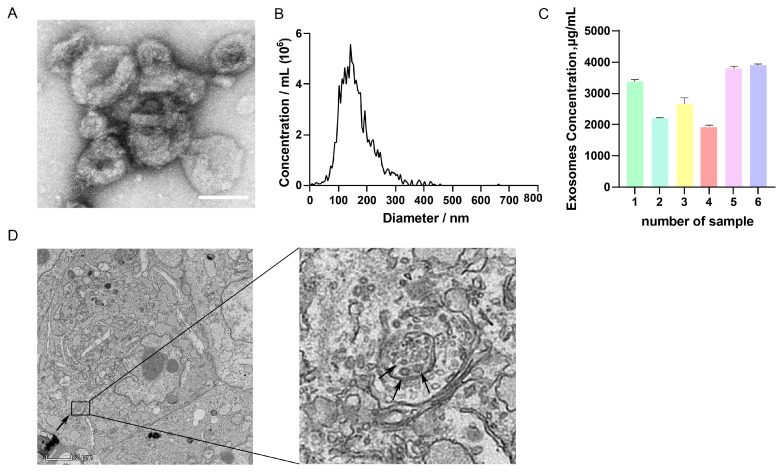
Characterization of GC-EXs. (**A**) TEM image of GC-EXs. Scale bar = 100 nm; (**B**) NTA to detect the particle size of EXs; (**C**) concentration analysis of BCA proteins in GC-EXs; (**D**) TEM image of GC section. The arrow on the left represents MVBs, the arrow on the right represents GC-EXs. Scale bar = 2 μm.

TEM analysis revealed that GC-EXs exhibited a characteristic cup-like morphology with a central depression (Figure 1A). NTA analysis displayed that the diameter of the EXs was largely within the range of 100–180 nm (Figure 1B), consistent with the particle size characteristics of EXs. BCA protein concentration analysis indicated that the concentration of exosomal protein was above 2000 μg/mL (Figure 1C). TEM analysis revealed the presence of many MVBs and EXs in GCs (Figure 1D). In summary, these results demonstrated that the extracted particles exhibited the basic characteristics of EXs and had a sufficiently high concentration to achieve satisfactory separation effects, inferring that they can be used for subsequent studies on EXs.

### 3.2. COCs Ingestion of GC-EXs

**Figure 2:**
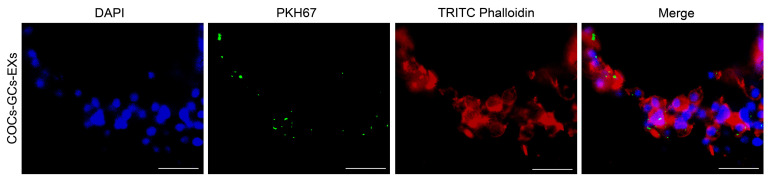
Internalization of GC-EXs by COCs was observed by laser confocal microscopy. DAPI: cell nucleus, PKH67: EXs, TRITC Phalloidin: cell membrane actin. Scale bar = 25 μm.

To determine the absorption rates of GC-EXs by COCs, EXs were labeled with PKH67, TRITC Phalloidin with cell membrane actin, while the cell nuclei were labeled with DAPI. Laser confocal microscopy showed the presence of green fluorescent EXs in cumulus cells surrounding COCs (Figure 2).

### 3.3. Effects of GC-EXs of Different Concentrations on Cumulus Expansion and Oocyte Maturation

**Figure 3:**
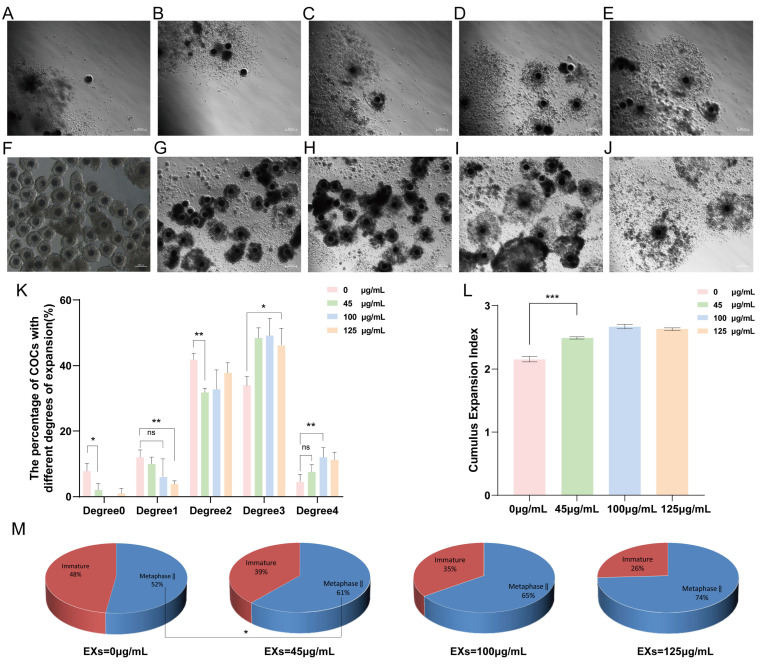
Effects of different concentrations of GC-EXs on the cumulus expansion and oocyte maturation. Scale bar = 200 μm. (**A**–**E**) Cumulus expansion degrees 0–4 for COCs, respectively; (**F**) COCs before in vitro culture; (**G**–**J**) COCs after treatment 42–44 h with 0, 45, 100, and 125 μg/mL GC-EXs, respectively; (**K**) distribution of the cumulus expansion degrees of COCs after treatment with different concentrations of GC-EXs; (**L**) the CEI after treatment with different concentrations of GC-EXs; (**M**) oocyte maturation rate after treatment with different concentrations of GC-EXs. ns *p* > 0.05, * *p* < 0.05, ** *p* < 0.01, and *** *p* < 0.001.

To determine the optimal concentration of GC-EXs in COCs, four concentration gradients were examined in this experiment. Specifically, 0 (DPBS), 45, 100, and 125 μg/mL GC-EXs were added to the oocyte maturation medium in vitro (Figure 3F–J). The COCs were randomly grouped and cultured in the medium. A total of 518 COCs were used in three replicates to assess the impact of different concentrations of EXs on cumulus expansion and oocyte maturation. As illustrated in the figure, the inclusion of EXs decreased the proportion of degrees 0–2 COCs, increased the proportion of degree 3 and 4 COCs (Figure 3K), promoted cumulus expansion (Figure 3L), and significantly enhanced the in vitro maturation rate of oocytes (Figure 3M). As anticipated, the introduction of 100 μg/mL EXs was more effective in promoting cumulus expansion and exerted a positive impact on the oocyte maturation rate. Therefore, we selected 100 μg/mL EXs for subsequent experiments.

### 3.4. Effects of 100 µg/mL GC-EXs on Oocyte Development

**Figure 4:**
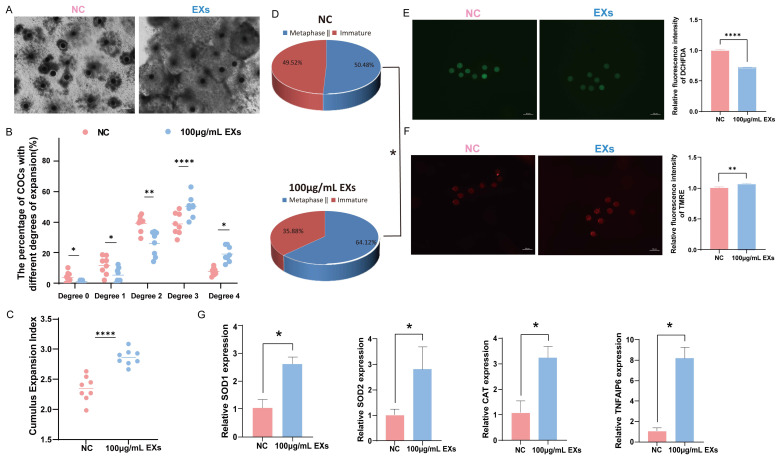
Effect of 100 µg/mL GC-EXs on oocyte development. Scale bar = 50 μm. (**A**) COCs after 42–44 h of treatment with DPBS and 100 µg/mL GC-EXs; (**B**) distribution of COC cumulus expansion degrees; (**C**) the CEI after treatment with DPBS and 100 µg/mL GC-EXs; (**D**) oocyte maturation rate; (**E**) the level of ROS in mature oocytes; (**F**) the level of MMP in mature oocytes; (**G**) expression of genes related to cumulus expansion and antioxidative stress. * *p* < 0.05, ** *p* < 0.01, and **** *p* < 0.0001.

To ensure the reliability of the data, eight replicates were constructed using 966 COCs to evaluate the impact of 100 µg/mL EXs on cumulus expansion and oocyte maturation rates. Notably, treatment with 100 µg/mL EXs consistently enhanced oocyte maturation and cumulus expansion (Figure 4A–D). Simultaneously, ROS and MMP staining assays were conducted on mature oocytes. The results exposed that the introduction of 100 µg/mL EXs effectively reduced the level of ROS in mature oocytes (Figure 4E) and increased the level of MMP (Figure 4F). Additionally, the total RNA was extracted from GCs and oocytes separately, and RT-qPCR was performed for detection. After treatment with 100 µg/mL EXs, the expression of genes related to cumulus expansion (TNFAIP6) was significantly higher in GCs, while genes related to antioxidative stress (SOD1, SOD2, CAT) were upregulated in oocytes compared to the control (Figure 4G).

### 3.5. Sequencing Results and Analysis

**Figure 5:**
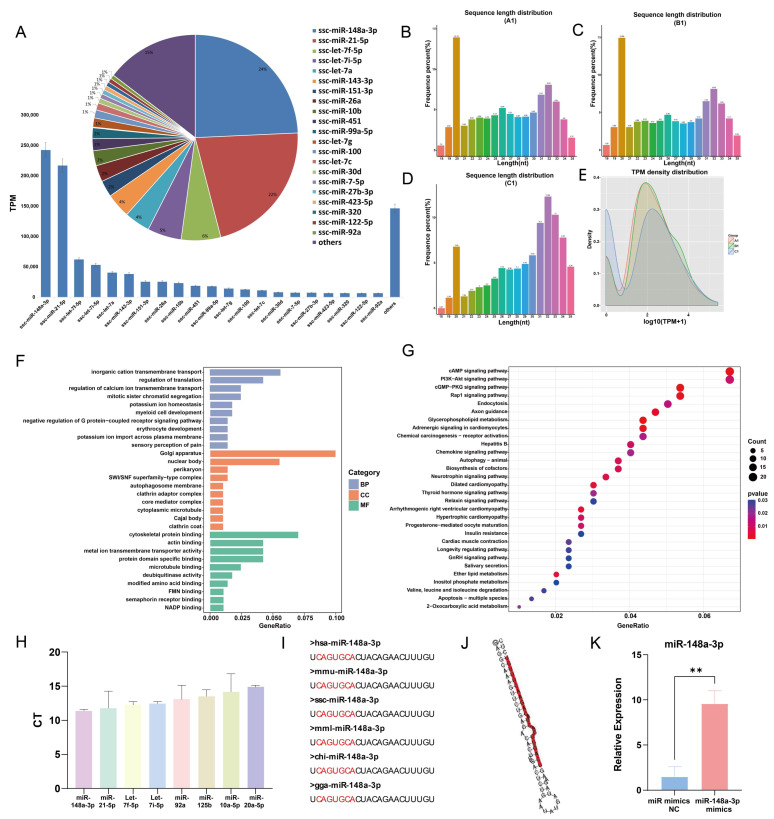
sRNA sequencing results and analysis. (**A**) Schematic of the top 21 TPM values of the sequencing results; (**B**–**D**) sRNA length distribution; (**E**) TPM density distribution map; (**F**) GO analysis; (**G**) KEGG analysis; (**H**) validation of the sequencing results by RT-qPCR; (**I**) conservativeness analysis of miR-148a-3p; (**J**) secondary structure of miR-148a-3p; (**K**) efficiency of overexpression miR-148a-3p mimics. ** *p* < 0.01.

A total of 272 miRNAs were identified in GC-EXs through sRNA sequencing. The top 21 miRNAs with the highest expression levels accounted for 85.35% of miRNA reads (Figure 5A). Typically, porcine sRNA ranges from 18–35 nt in length, with the majority of miRNAs being 20–23 nt long (Figure 5B–D). In the present study, the sRNA lengths were predominantly distributed around 20 nt. In addition, TPM densities were analyzed, unveiling that the distribution of gene expression was similar in the three samples, thereby indicating that the samples were reproducible (Figure 5E). The target genes of the top 21 miRNAs with the highest expression levels were predicted and analyzed using GO and KEGG analyses. The GO enrichment analysis determined that transmembrane transport and translation regulation were the most enriched biological processes. Regarding cellular components, they were primarily enriched in the nucleus, endoplasmic reticulum, and Golgi apparatus. In terms of molecular functions, the miRNAs were mainly concentrated in cytoskeletal binding, actin binding, and so on (Figure 5F). The results of the KEGG enrichment analysis suggested that the target genes primarily regulated signaling pathways involving PI3K-AKT, Rap1, GnRH, glycerophospholipid metabolism, and progesterone-mediated oocyte maturation (Figure 5G). These pathways are closely associated with follicular development and function. To validate the accuracy of the sequencing results, RNA from GC-EXs that were not sequenced was extracted. We randomly selected four miRNAs with high expression (miR-148a-3p, miR-21-5p, let-7f-5p, let-7i-5p) and four miRNAs with low expression (miR-92a, miR-125b, miR-10a-5p, miR-20a-5p). The sequencing and RT-qPCR results (Figure 5H) demonstrated consistent expression patterns of the miRNAs, indicating the sequencing data can reflect the expression pattern of miRNAs in GC-EXs.

Interestingly, miR-148a-3p had the highest expression level (24.35%) in GC-EXs, highlighting its potential role in the functional regulation of GC-EXs. The previous studies conducted by our research group have identified that miR-148a-3p is differentially expressed in FF during the mature and immature stages. The conservation of mature miR-148a-3p was observed by comparing its sequences across different species (Figure 5I). Additionally, miR-148a-3p has been detected in extracellular vesicles secreted by fallopian tube stem cells and may have a role in oocyte maturation [48]. Therefore, we hypothesized that miR-148a-3p plays a significant role in GC-EXs. To validate this hypothesis, 40 nM miR-148a-3p mimics were transfected, and the overexpression efficiency was verified. The expression level of miR-148a-3p was significantly increased after transfection compared to the NC group (Figure 5K). This indicates that the overexpressed miR-148a-3p model was successfully constructed.

### 3.6. Effects of miR-148a-3p on Oocyte Development

**Figure 6:**
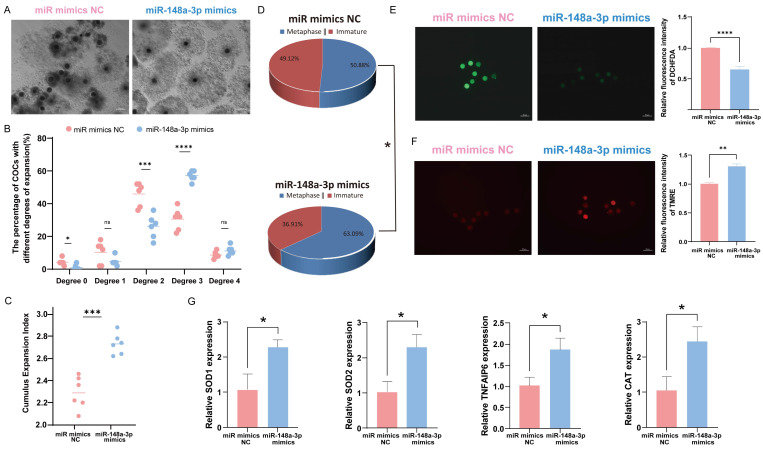
Effect of overexpression of miR-148a-3p on oocyte development. Scale bar = 50 μm. (**A**) COCs after 42–44 h of treatment with NC and miR-148a-3p; (**B**) distribution of COCs expansion degrees; (**C**) the CEI after treatment with NC and miR-148a-3p; (**D**) oocyte maturation rate; (**E**) the level of ROS in mature oocytes; (**F**) the level of MMP in mature oocytes; (**G**) expression of genes related to cumulus expansion and antioxidative stress. ns *p* > 0.05, * *p* < 0.05, ** *p* < 0.01, *** *p* < 0.001, and **** *p* < 0.0001.

To investigate the role of miR-148a-3p in oocytes, we divided 600 COCs into six groups and assessed the impact of miR-148a-3p mimics on cumulus expansion and oocyte maturation. The results indicate that overexpression of miR-148a-3p enhances the maturation rate of oocytes and promotes cumulus expansion (Figure 6A–D). ROS and MMP staining of mature oocytes demonstrated that miR-148a-3p overexpression reduced ROS (Figure 6E) and increased the level of MMP (Figure 6F) consistent with the effect of GC-EXs. The RNA was extracted from GCs and oocytes separately, and RT-qPCR was performed for detection. After transfection with miR-148a-3p mimics, the expression of TNFAIP6, a gene related to cumulus expansion, significantly increased in GCs. Additionally, the expression of genes related to antioxidative stress, including SOD1, SOD2, and CAT, significantly increased in oocytes compared to the control group (Figure 6G).

### 3.7. Prediction and Verification of miR-148a-3p Target Gene

**Figure 7:**
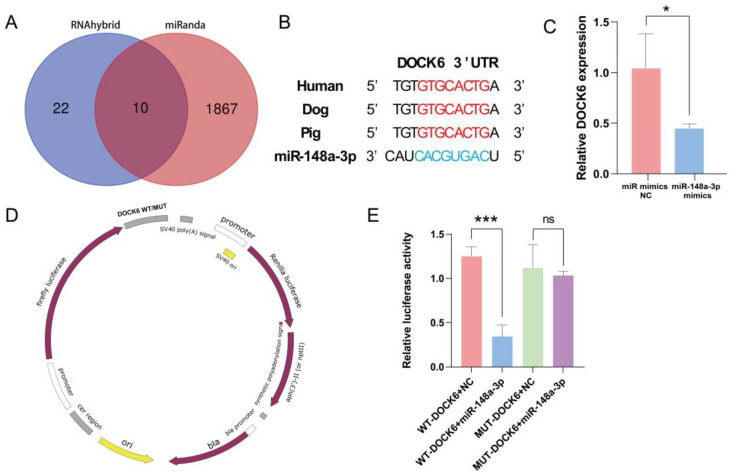
miR-148a-3p target gene prediction and validation, (**A**) Target gene prediction; (**B**) DOCK6 conservation analysis; (**C**) DOCK6 expression after overexpression of miR-148a-3p; (**D**) dual-luciferase reporter plasmid; (**E**) dual-luciferase reporter gene validates direct targeting of miR-148a-3p to DOCK6. ns *p* > 0.05, * *p* < 0.05, and *** *p* < 0.001.

The miRNA target gene prediction software, miRanda (v.3.3), and RNAhybrid (v.2.1.2) were used to predict ten potential target genes of ssc-miR-148a-3p (Figure 7A). Reviewing the literature to analyze its function unveiled that DOCK6 plays an instrumental role in regulating cell cycle, proliferation, migration, and apoptosis. Therefore, DOCK6 was initially selected as a candidate target gene for subsequent analysis. The 3′UTR sequence of DOCK6 is conserved in mammals, including humans, dogs, and pigs (Figure 7B). Notably, the overexpression of miR-148a-3p significantly downregulated DOCK6 expression (Figure 7C). Furthermore, dual-luciferase reporter plasmids of pmirGLO-DOCK6-3′UTR-WT and pmirGLO-DOCK6-3′UTR-MUT were synthesized (Figure 7D). Specifically, co-transfection with WT-DOCK6-3′UTR resulted in a significant decrease in luciferase activity, whereas co-transfection with MUT-DOCK6-3′UTR did not result in a significant difference in luciferase activity (Figure 7E). Taken together, these results indicate a targeting relationship between DOCK6 and miR-148a-3p.

## 4. Discussion

Mammalian follicular development, maturation, and ovulation are complex biological processes that involve the regulation of numerous factors [49]. The proliferation and differentiation of granulosa cells have been reported to play an essential role in follicular recruitment, selection, and ovulation. Additionally, the apoptosis of granulosa cells can induce follicular atresia [50,51]. Therefore, the signal exchange between oocytes and granulosa cells is crucial [52]. The proper development of follicles has a direct impact on animal reproductive performance and economic efficiency. Consequently, it is crucial to identify efficient methods to attenuate follicular atresia and increase the utilization efficiency of follicles and oocytes.

In recent years, exosomes in follicular fluids have been established to play a critical role in follicular development. However, the environment in which follicular fluid exosomes are found is complex, and their specific sources and regulatory mechanisms are yet to be fully understood. Our study demonstrated that porcine granulosa cells can secrete exosomes, and their morphology and size [53], as well as their levels [54], are consistent with the observations of previous studies. Furthermore, our results demonstrated that GC-EXs may be absorbed by COCs. Nonetheless, while our study determined that COCs can absorb EXs, it did not conclusively establish that EXs can penetrate the zona pellucida to be taken up by oocytes and play a direct role in regulating oocyte development. This is in line with the findings of Hung et al. [55]. Indeed, earlier studies have not established that EXs can cross the zona pellucida, warranting further investigation.

Cumulus cells are a type of cell formed by the progressive differentiation of GCs following the formation of the follicular cavity. They surround the oocyte in a columnar shape [56]. Of note, the expansion of cumulus cells promotes oocyte maturation [57]. Hung [55] isolated EVs in bovine FF and noted that they can induce cumulus expansion in mice and cattle. Herein, the addition of GC-EXs significantly improved the cumulus expansion index, promoted cumulus expansion, and improved the maturation rate of oocytes. However, Matsuno et al. [58] showed that the addition of porcine FF-EVs did not effectively promote porcine cumulus expansion. This discrepancy in GC-EXs and FF-EVs may be attributed to their distinct sources and the primary substances involved. Technical differences, such as the methods used to separate EVs, might also have contributed to this variation, necessitating further discussion.

Cells experience oxidative stress when intracellular levels of ROS exceed the internal antioxidant capacity [59,60]. In vitro, cultured oocytes are particularly susceptible to damage by oxidative stress due to a deficiency in antioxidants and ROS accumulation in the environment, leading to mitochondrial damage, DNA damage, apoptosis, and aberrant meiosis [61]. Mitochondria play a vital role in regulating the quality of oocytes [62]. Indeed, they serve as primary energy-producing organelles and are also a source of reactive oxygen species. Moreover, mitochondria store electrochemical potential energy in the inner mitochondrial membrane during energy production. This creates an asymmetric distribution of ionic concentrations, resulting in MMP on both sides of the membrane [63]. The stabilization of the MMP is crucial for maintaining the normal function of oocytes [64]. Abnormal mitochondrial function and insufficient energy reserves can drive abnormal meiosis in oocytes [65]. The addition of antioxidants to oocyte culture in vitro has been shown to exert a positive effect, improving oocyte maturation rates by attenuating oxidative stress and restoring impaired mitochondrial performance. In the present study, the addition of GC-EXs reduced ROS levels in oocytes, increased the mitochondrial membrane potential, and optimized antioxidant capacity [66,67,68]. As a result, the developmental level and quality of oocytes significantly improved. Recent studies have evinced that EXs can serve as carrier systems for loading drugs and act as antioxidants [69,70]. Ascribed to their stability, versatility, and ability to specifically target receptors, EXs have been considered novel diagnostic and therapeutic tools in reproductive biology [71]. The results of our study also corroborated that exosomes can act as antioxidants and be applied to in vitro oocyte cultures in the future.

MiR-148a-3p is a member of the miR-148/152 family and comprises miR-148a, miR-148b, and miR-152 [72]. In a previous study, the expression profiles of oocyte miRNAs in follicles of different sizes were characterized [73]. The study found that miR-148a-3p was highly expressed and may play a role in oocyte development. In this study, miR-148a-3p, identified through sequencing of GC-EXs, may play a significant role in oocyte development. The transfection of miR-148a-3p mimics resulted in a significant increase in the developmental level and quality of oocytes compared to the control group, consistent with the effect of adding GC-EXs. This observation validates that miR-148a-3p is a GC-EX-like functioning factor and plays a regulatory role in oocyte development. However, it should be noted that our study only involved miRNA overexpression treatment and lacked inhibition experiments; the validation of miRNA function was not comprehensive, which is a limitation of this study. In the future, a more in-depth study using the microinjection method could be considered. There is mounting evidence that suggests that exosomal miRNAs can be used as target carriers for the treatment of a large number of diseases [74]. However, miRNAs are not randomly incorporated into exosomes but are selectively wrapped [75] The mechanism by which miR-148a-3p is selectively wrapped is not yet understood, and further exploration is required.

Dedicator of cytokinesis 6 (DOCK6) is an atypical ornithine nucleotide exchange factor that activates Rho GTPase, thereby mediating cell morphology, proliferation, apoptosis, adhesion, and migration [76]. It also regulates progesterone synthesis [77]. This study identified DOCK6 as a potential target gene of ssc-miR-148a-3p using miRanda and RNAhybrid. Their targeting relationship was confirmed by dual-luciferase reporter assay and RT-qPCR. Our results revealed that miR-148a-3p regulates the development of COCs by mediating DOCK6. However, further research is warranted to determine the regulatory effect of DOCK6 on cellular functions via the regulation of hormone synthesis.

## 5. Conclusions

In summary, this study confirmed that porcine GCs can secrete EXs. These GC-EXs can be subsequently taken up by COCs, significantly promote oocyte cumulus expansion, increase oocyte maturation rate, decrease ROS levels, and increase MMP levels by increasing cumulus expansion and the expression of genes related to oocyte antioxidative stress (SOD1, SOD2, CAT, and TNFAIP6). Additionally, RNA was extracted from GC-EXs for sRNA sequencing. The sequencing data indicated that miR-148a-3p may be the key miRNA that plays a role in GC-EXs. In addition, miR-148a-3p mimics were transfected into COCs. Compared to the control group, the transfection of miR-148a-3p improved the development level and quality of oocytes, yielding effects comparable with the addition of EXs. Bioinformatics prediction, dual luciferase reporter gene assay, and RT-qPCR validated DOCK6 as the target gene of miR-148a-3p. The study revealed that GC-EXs regulate DOCK6 through miR-148a-3p, thereby affecting COCs development. In conclusion, this study can serve as a reference for enhancing the level of porcine oocyte development. These findings hold significant clinical applications in in vitro embryo production and cloning technology. Furthermore, female ovarian disease is typically caused by impaired oocyte development and disturbances in the regulation of reproductive hormones, and our results confirm that GC-EXs can improve oocyte development. Therefore, we speculated that GC-EXs have potential clinical applications in the treatment of female ovarian diseases.

## Data Availability

The data are contained within the article.

## Supplementary Materials

The following supporting information can be downloaded at: https://www.mdpi.com/article/10.3390/antiox13030348/s1, Table S1: Primer sequences used for RT-qPCR.
