# Supplementary material for: Porcine Granulosa-Cell-Derived Exosomes Enhance Oocyte Development: An In Vitro Study
Source: Antioxidants (Basel). 2024 Mar 14;13(3):348. doi: 10.3390/antiox13030348 (PMC10968481; doi:10.3390/antiox13030348)
Supplement: Supplementary file 1 [file antioxidants-13-00348-s001.zip › antioxidants-2897889-supplementary.pdf]

Table S1. Primer sequences used for RT-qPCR

| Genes | Type | Primer Sequences (5′ –3′ ) | Accession No. |
|---|---|---|---|
| SOD1 | F | GTGGCCACTGTGTACATCGA | NM_001190422.1 |
| | R | CCCAATTACACCACAGGCCA | |
| SOD2 | F | ATCAACGCCCAGATCATGCA | NM_214127.2 |
| | R | GCCTCCACCGTTGAACTTCA | |
| CAT | F | GCCTGTGTGAGAACATTGCG | NM_214301.2 |
| | R | AAGTGAGATCCAGCCTGCAC | |
| TNFAIP6 | F | GCGTGTACCTCAGAGAAGCA | NM_001159607.1 |
| | R | GACGGCCACCTTCGTATTCA | |
| GAPDH | F | TCGGAGTGAACGGATTTGGC | NM_001206359.1 |
| | R | TGCCGTGGGTGGAATCATAC | |
| U6 | F | GGAACGATACAGAGAAGATT AGC | EU520423.1 |
| | R | TGGAACGCTTCACGAATTTGC G | |
| miR-148a-3p | F | AACACGTGTCAGTGCACTAC AGA | NR_038485.1 |
| | R | ATCCAGTGCAGGGTCCGAGG | |
